# No Direct Association Between Asthma and the Microbiome Based on Currently Available Techniques

**DOI:** 10.1097/MD.0000000000000199

**Published:** 2014-12-12

**Authors:** Josef Yayan

**Affiliations:** Department of Internal Medicine, Division of Pulmonary, Allergy and Sleep Medicine, Saarland University Medical Center, Homburg/Saar, Germany.

## Abstract

Current uses of culture-independent tools in previous studies have shown a significant relationship between microbiota and asthma. Although these studies are relatively new, there is also evidence of the possibility of new therapeutic strategies for the treatment or prevention of asthma.

This article retrospectively examines the possible association between microorganisms and asthma.

Data on all patients with different types of asthma were collected from hospital charts from the Department of Internal Medicine, Saarland University Medical Center, Germany, within the study period of 2011 to 2012.

The tracheal secretions of asthmatics obtained by bronchoalveolar lavage, bronchial aspirates through flexible bronchoscopy, and directly in sputum were examined microbiologically for microorganisms. Thirty-one (10.47%, 95% CI, 6.98–13.96) of a total of 296 patients were found to have asthma microorganisms in their airways. We could not establish a causal relationship between microorganisms and asthma based on the results of our study (*P* = 0.893). Additionally, acute respiratory infections did not affect the microbiological colonization in asthmatics’ airways (*P* = 0.472).

We were unable to find a direct association between asthma and the microbiome based on existing diagnostic techniques.

## INTRODUCTION

Asthma is a chronic inflammatory disease of the respiratory tract. Predisposed individuals suffer recurrent episodes of symptoms such as wheezing, cough, and dyspnea due to bronchial obstruction. The airway obstruction is reversible, either spontaneously or through medical treatment. Inflammation causes an increase in the sensitivity of the airway to a variety of stimuli. This increase in sensitivity is referred to as bronchial hyperreactivity.^1^ Asthma was traditionally understood to be a misdirected immune reaction to environmental influences, and thus believed to be caused by the inhalation of allergens. The latest studies, however, have challenged this outlook on asthma by offering new insights into the pathophysiological relationship between microbial exposures, early as well as successive infections, and the development of asthma. Furthermore, recent scientific works have pointed out that the lung microbiome is different in asthma patients than in healthy subjects. Previously, the lungs of healthy subjects were considered aseptic.^2–4^ However, it is not clear whether there is a permanent or temporary bacterial colonization in the lungs. Such conclusions were due to the absence of microorganism growth on culture media, which we know detect only a few pathogenic germs. Altogether, the experimental, epidemiological, and clinical studies establish the contemporary hypothesis that modifications in the native lung microbiota can be a predisposing factor for the development of asthma. These new findings expand the common explanations of asthma enormously. The recent insights into the complicity of the lung microbiota with the emergence of asthma now raise new questions. We conducted the present investigation to better understand all the possible types of asthma caused by a spectrum of possible pathogenic microorganisms, which we want to identify by examining the respiratory flora in asthmatic patients. Therefore, we collected data on all patients with various types of asthma classified as allergic, non-allergic, mixed, or unspecified forms according to the International Classification of Diseases (ICD) and who were examined for microbiome from the hospital database at the Department of Internal Medicine, Saarland University Medical Center, Germany. The variety of tested organisms consisted of bacteria, viruses, and fungi in the airways of different types of asthmatics. Respiratory infections in asthmatics were diagnosed after culturing bronchial mucus on agar obtained either through extraction by bronchoalveolar lavage, bronchial aspirate, or throat swabs or through sputum production. The aim of this study is to clarify which of those methods better detects pathogens in the sputum of bronchial asthma patients. This study was designed to clarify whether pathogens detected were infection-associated or appeared during intervals free from asthma attacks. Only once we have identified the causes involved in each case of asthma can we develop appropriately tailored therapies for asthmatics.

## MATERIALS AND METHODS

### Patients

This study retrospectively examined the pathogen spectrum in asthma patients collected from hospital chart data at the Department of Internal Medicine of the Saarland University Medical Center in the study period from 2011 to 2012. Asthma has been defined as a chronic inflammatory disease of the respiratory tract characterized by bronchial hyperreactivity and variable airway obstruction, which are either partially or completely reversible. Asthmatic symptoms were classified as paroxysmal dyspnea under the clinical presentation of expiratory stridor, cough-variant asthma, and wheezing. The diagnosis of manifested asthma was made based on patients’ medical history, clinical examinations, and lung function with bronchospasmolysis. Asthma was classified in each case according to the latest edition of the ICD (J45.0–J45.9) from 2011 to 2012. We compared the lung microbiome in predominantly allergic asthma (J45.0), non-allergic asthma (J45.1), mixed asthma (J.45.1), and unspecified asthma patients (J45.9). The study population was mixed in terms of age in all 4 study groups. It included unselected asthmatics both during asthma attack periods and attack-free intervals.

The goal of this study was to determine whether the detection of pathogens was infection-associated in these 4 types of asthma. The indication for the microbiological examination was performed either explicitly because of a suspected respiratory infection or otherwise routinely. Microbiological testing was explicitly performed for pathogenic microbes, viruses, fungi, legionellae, mycobacteria, nocardiasis, actinomycetales, pneumocystis jiroveci (carinii), and polymerase chain reaction (PCR) (eg, atypical pneumonia pathogens, mycobacteria). Secretions from the mouth-nasal cavity and trachea were obtained differently depending on the particular case; the commonly applied methods were bronchoalveolar lavage, tracheal secretions, throat swabs, and sputum. The bronchoalveolar lavage was applied in the context of a bronchoscopy. In this case, about 20 mL of 0.9% saline solution was instilled under local anesthesia and aspirated through the fiber-optic bronchoscope again. The aspirate thus obtained was deposited into 3 different sterile specimen traps (tracheal suction set, Dahlhausen Medizintechnik, Köln, Germany). Tracheal secretions were also collected by fiber-optic bronchoscopy through aspiration into a sterile specimen trap (tracheal suction set, Dahlhausen Medizintechnik). The throat swab was collected with a commercial cotton swab transport system (Copan Italia, Brescia, Italy) by rotating the swab under slight pressure on the palatal arch of asthma patients. The sputum was recovered by expectoration into a 30 mL sterile sputum collection tube (Salivette, SARSTEDT, Nümbrecht, Germany) and sent to the laboratory for analysis.

After collecting samples of sputum, tracheal, and bronchial secretions, the test samples were transported in suitable containers to the Institute of Microbiology and Virology. After propagating the sputum in a sterile Petri dish and testing it against a dark background, the macroscopic evaluation was performed to determine whether it was slimy, purulent, or bloody. Thereafter, bronchial secretions and pus constituents of the saliva were separated with a needle and an eye. Supportive shares were used for microscopic examination. Microscopic examination was done after Gram staining in 80- to 1000-fold magnification of at least 5 visual fields according to the criteria of Bartlett. In typical morphology and a larger number of suspected diagnosis of the pathogen in the microscopic bacteriological examination according to the microbiological infectiological quality standards was expressed. The semi-quantitative determination of the squamous epithelia and granulocytes and of microorganisms was performed. Subsequently, 3 solid culture media were applied to cultivate the most common aerobic, fast-growing microorganisms as a base culture approach. Columbia Agar with 5% sheep blood (Oxoid, Wesel, Germany) agar (Becton Dickinson, Heidelberg, Germany) was used and incubated at 37 °C for 24 to 48 hours as a general culture medium for the growth and discovery of *Streptococcus pneumoniae, Streptococcus pyogenes*, *Staphylococcus auereus, Escherica coli*, and *Shigella flexner*. Gassner agar (water-blue metachrome–yellow lactose agar) (Oxoid) operated to detect *Enterobacteriaceae.* The tested *Enterobacteriaceae* were *E coli, Shigella, Klebsiella, Proteus mirabilis, Enterobacter* sp., *Citrobacter* sp., *Serratia marcescens, Salmonella,* and *Yersinia.* A medium (Wilkins-Chalgren Anaerobe Agar, Oxoid) containing Columia agar nalidixic acid for the general growth of anaerobes was used for antimicrobial susceptibility testing.

The chocolate agar (Oxoid Blood Agar Base No. 2, Wesel, Germany) was used as a variant of blood agar, in which by brief heating of the agar at 80 °C, the lysis of erythrocytes was achieved. As a result, hemin (“Factor X”) and nicotinamide adenine dinucleotide (“Factor Y”) were released into the agar and metabolized by bacteria; the hemolytics themselves were absent. Cooking blood plates were sometimes incubated at an elevated carbon dioxide tension, as capnophile germs, like all *Haemophilus influenca*, *Brucella*, and *Campylobactor*, multiply so much better.

MacConkey agar (Oxoid) was used as a selective medium to detect gram-negative bacteria.

This Sabouraud agar (Oxoid) worked for the cultivation and differentiation of fungi.

PCR was used to detect adenovirus; coronavirus; coxsackie ECHO virus; cytomegalovirus; Epstein–Barr virus; Herpes simplex; Influenza viruses A, B, and H1/N1; parainfluenzas 1 to 3; respiratory synctial virus; rhinovirus; enterovirus; and varicella-zoster virus.

The mechanical properties of the lungs and the lung volume were measured using body plethysmography (JAEGER, MasterScreen Body, Germany). The inclusion criteria for this study were all patients with asthma whose tracheal secretions were examined microbiologically for pathogenic germs. All patients with asthma who were not subjected to a microbiological test were excluded from this study.

We considered the following parameters of body plethysmography for this study: the forced expiratory volume in the first second (FEV1), the inspiratory vital capacity (VCin), the total lung capacity (TLC), and the Tiffeneau index (FEV1%VCin). The measurement of exhaled nitric oxide (FeNO) expressed as parts per billion (ppb) was determined by a chemiluminescence analyzer as a flow control in asthmatics. Blood gas analysis was performed by obtaining blood from the earlobe of each patient about 10 minutes after rubbing it with a circulation-raising ointment (Finalgon Cream, nonivamide/butoxyethyl nicotinate, Boehringer Ingelheim Pty Limited, Ingelheim am Rhein, Germany): oxygen, carbon dioxide, and pH scale were all measured with the blood gas analyzer ABL90 FLEX (Radiometer Ltd., Willich, Germany). The reference range after blood gas analysis was 7.37 to 7.42 for the pH scale, 75 to 90 mm Hg for oxygen, and 37 to 43 mm Hg for carbon dioxide. The prick test was carried out as part of the allergic asthma diagnosis. In this common test, liquid allergens such as dust mites, grass pollen, animal hair, epithelia, etc. were introduced into the surface layer of the skin by means of scratching. After a waiting time of 20 minutes, the skin's reaction was measured with the template provided.

The total immunoglobulin E (IgE) in human serum and plasma was determined as an aid in the diagnosis of allergic diseases. The blood samples for IgE were collected using the SARSTEDT serum Monovette 4.7 mL (brown top) blood collection system with a multifly blood collection needle. Electrochemiluminescence immunoassay (ECLIA) was performed to determine IgE on Elecsys 2010 and cobase 411 immunoassay analyzers (Roche Diagnostics Ltd., Mannheim, Germany). The normal reference range for IgE was defined as 0 to 158 IU/mL. The C-reactive protein (CRP) in human serum and plasma was measured quantitatively after sample collection in lithium heparin SARSTEDT Monovette 4.7 mL (orange top) using a standard immunoturbidometric assay on the COBAS INTEGRA system (the normal value is less than 6 mg/L). Flow cytometry was used to determine differential white blood cell counts such as neutrophils (normal range 50–75%) and eosinophils (normal range 0–5%), which were collected in EDTA Monovette 2.7 mL (SARSTEDT, Nümbrecht, Germany). The leukocyte count (normal range 4000–10,000/μL) in the blood was determined generally as a routine part of small or large blood counts after collection in EDTA Monovette 2.7 mL by flow cytometry. We examined long-term oxygen therapy (oxygen <75 mm Hg, carbon dioxide >50 mm Hg) as a predisposing factor for bacterial colonization in asthma. Allergic conditions have been studied as factors favoring the colonization of pathogens in bronchial asthma. These included allergic rhinitis, allergic conjunctivitis, atopic dermatitis, food allergy, and drug allergies. We analyzed the cases for selected comorbidities—chronic obstructive pulmonary disease (COPD), gastroesophageal reflux disease, obesity, and depression—not only as predisposing factors for the development of asthma, but also for bacterial colonization in such patients. The length of hospital stay was compared between patients with different forms of asthma with and without the detection of infection. Saarland's Institutional Review Board approved the study. Due to the retrospective nature of the study protocol, the Medical Association of Saarland's Institutional Review Board waived the need for informed consent. Written informed consent was waived because the study used a retrospective analysis of patients’ medical records. All patients’ data were anonymized prior to analysis.

### Statistical Analysis

The data were expressed in proportion, mean, and standard deviation wherever appropriate. We calculated 95% confidence intervals (CIs) for the total number of patients with asthma with microbial attacks. The chi-square test for 4 independent standard normal variables of 2 probabilities was used to compare the association between the microbiome and different types of asthma. Fishers exact test for 4 variables of 2 probabilities was used to evaluate sex differences, the association of infection, diagnostic tools for bacteria, viruses, fungi, the differentiation of microbes, prick tests, food and drug allergies, and allergic comorbidities in different types of asthma. One-way analysis of variance (ANOVA) for independent samples was performed to compare age differences, blood tests, lung function tests, FeNO, blood gas analysis, and the duration of hospital stay among all asthma groups. All tests were expressed as 2-tailed, and a *P* value of <0.05 was considered statistically significant.

## RESULTS

In our hospital database, we found 352 patients with different types of asthma who had been treated at the Department of Internal Medicine, University Hospital of Saarland, Germany, during the study period of 2011 to 2012. A total of 296 patients with asthma met the inclusion criteria for this trial. A total of 31 (10.47%, 95% CI, 6.98–13.96) patients with asthma were infested by germs microbiologically detected in their tracheal secretion. The most patients in this group were diagnosed with non-specified asthma, followed by allergic asthma, and the same-sized small groups of non-allergic and mixed asthmas (Table 1). Following the results of this study, we could not find any evidence of microorganisms as a causal source of asthma (*P* = 0.893, Table 1). Acute infection thus did not play a role in bacterial colonization in the respiratory tract of asthmatics (*P* = 0.472). One of the smallest groups in this study, mixed asthma, was the most comparatively prone to microbial attacks caused by acute infections. In contrast, acute infections did not play a significant role in allergic asthmatics, the second-largest group with microbial contamination in the respiratory tract (*P* = 0.472). A statically significant difference was found between the 4 groups in terms of their age distribution (*P* = 0.034). There were relatively more women affected by germs, without statistical significance (*P* = 0.4) 111, except in the group with allergic asthma. Diagnostic tools did not have any influence on the microbiological results in asthmatics (Table 1). A comparison of the different species showed an increased incidence of individual microbes, such as *Staphylococcus aureus*, *Respiratory synctial virus*, and *Candida albicans,* but without statistical significance (Table 2). The comparison of the blood test, lung function test, FeNO, and blood gas analysis results revealed no significant difference between the 4 asthma groups (Table 3). Skin allergy testing presented various medical diagnoses of allergies in all groups of asthma patients (Table 4). No difference was found between the groups when comparing the allergens, non-allergic asthmatics were allergic to adhesive, although most asthmatics had no plaster allergy in this study (*P* = 0.033). The comparison of food and drug allergies between groups was without statistical significance (Table 5). Allergies and various comorbidities investigated in this study showed no statistical differences between the 4 asthma groups (Table 6).

**TABLE 1:**
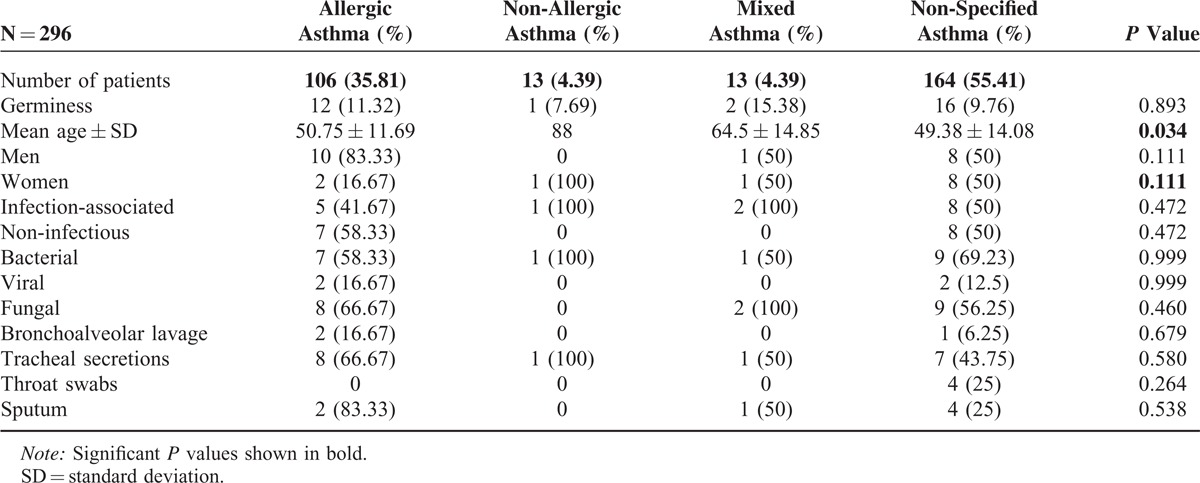
Demographic Data and Investigation Tools of the Microbiome in Different Types of Asthma

**TABLE 2:**
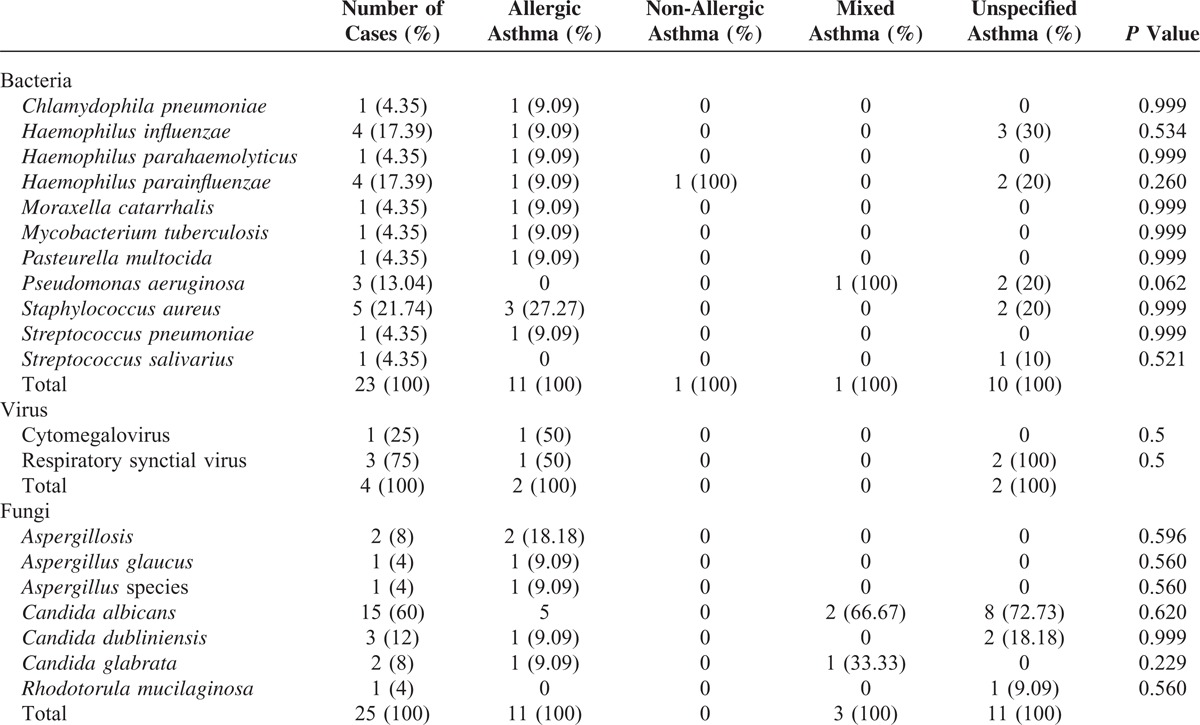
Comparison of the Different Microbe Species in Patients With Various Types of Asthma

**TABLE 3:**
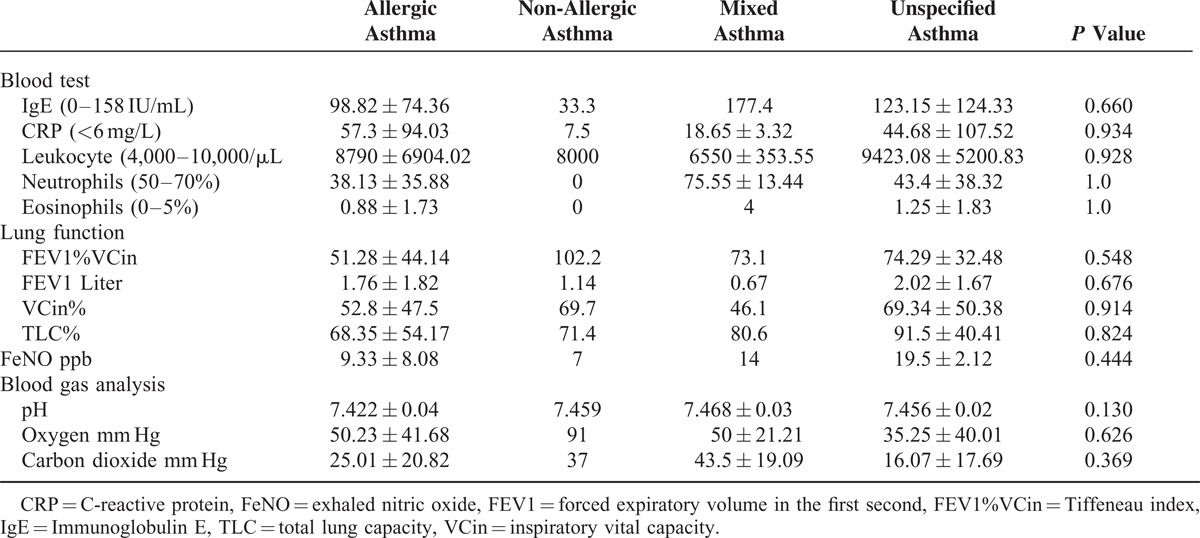
Comparison of the 4 Asthma Groups’ Blood Test, the Lung Function Test, Exhaled Nitric Oxide, and Blood Gas Analysis Results

**TABLE 4:**
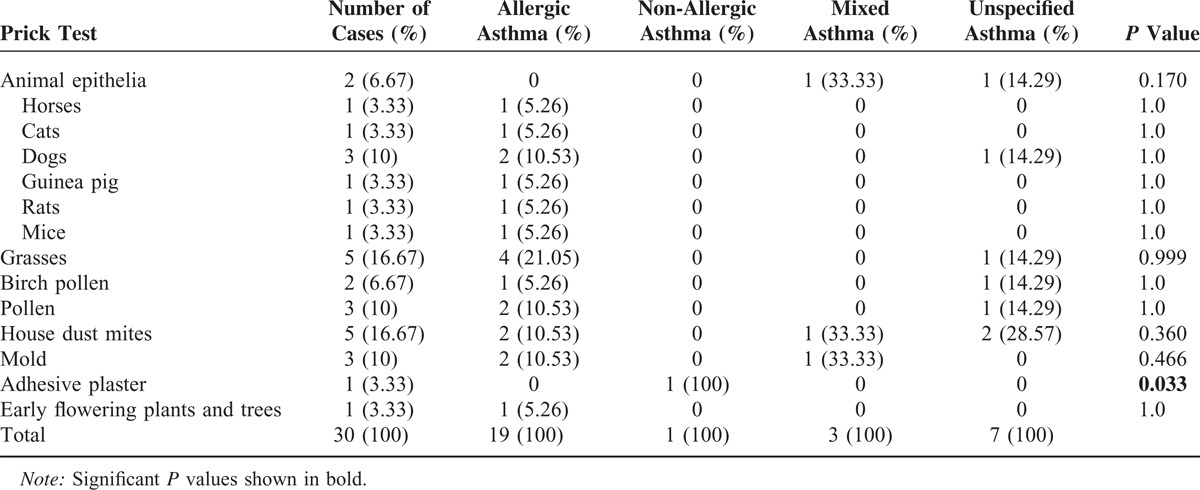
Different Allergens Detected in the 4 Asthma Groups Through Skin Prick Tests

**TABLE 5:**
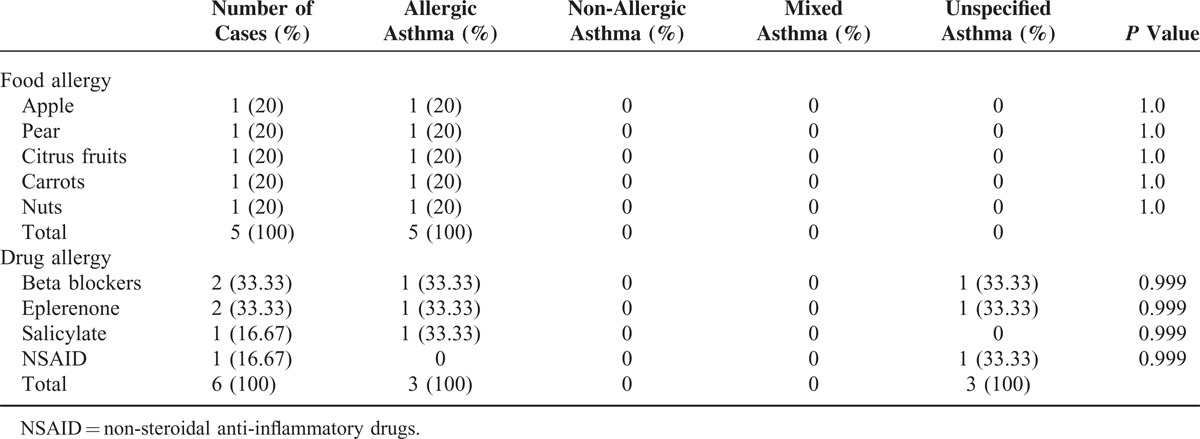
Comparison of Asthma Groups’ Food and Drug Allergies

**TABLE 6:**
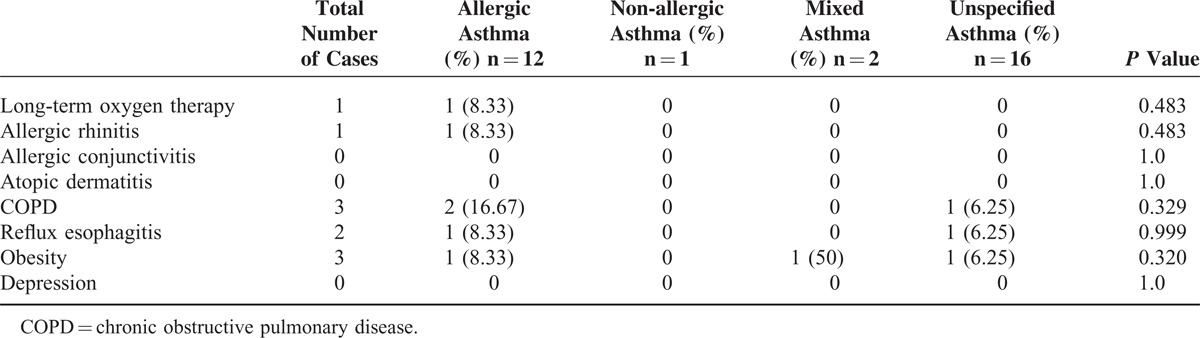
Comparison of Asthma Groups’ Allergic and Various Comorbidities

The duration of hospital stay was 11 ± 8.8 days in the allergic asthma group, 13 in the non-allergic, 11.5 ± 3.54 in the mixed, and 6.5 ± 6.87 in the non-specified, which is not a statistically significant difference (*P* = 0.407).

## DISCUSSION

Based on the present findings, we were unable to identify microbiological colonization in the airways of most asthmatics in this study population. The detection of pathogenic agents occurred in about 10% of asthmatics in this study. Therefore, there appeared to be no direct correlation between asthma and the microbiome. The evidence of germs occurred mainly in the context of acute respiratory infections, without statistical significance, in asthmatics in this trial. The pathogenesis of asthma is likely varied. A combination of genetic polymorphisms and environmental factors are suspected to lead to the development of asthma.^4^ Current data on the microbiome of the respiratory tract have demonstrated noteworthy differences between the number and variety of the microbial community in healthy subjects and asthmatics.^5^ Recent advances in the cultivation of germs and the establishment of new molecular techniques have contributed to the identification of microorganisms.^6^ New findings support the assumption of a connection between diverse microbial exposure in early life and the development of atopic diseases. An early colonization of the respiratory tract early in life increases the risk for the later development of asthma. Bacterial colonization of the respiratory tract appears to affect the course of asthma.^6^ There is convincing evidence that previous human rhinovirus and *Respiratory syncytial virus* infections are associated with an increased risk for developing asthma.^5^ We found respiratory viruses in the airways of only a few asthmatic cases in our study, so we were not able to make any connection between respiratory viruses and asthma from the data obtained in our study, although we did not evaluated earlier childhood infections with respiratory viruses in our study population.

Recent studies have demonstrated that the colonization of 2 unrelated species of atypical bacteria, *Mycoplasma pneumoniae* and *Chlamydophila pneumoniae*, in the respiratory tracts of most people contributes to the development of asthma.^5,7,8^ As is well known, acute respiratory infections favor the exacerbation of asthma.^7,8^ Despite a relatively large number of publications about the relationship of these 2 organisms and asthma, clear evidence about the link between these germs and asthma is still lacking.^8^ Although we were able to detect *C pneumoniae* by bronchoalveolar lavage in a patient with allergic asthma in an infection-free period, we were unable to identify this germ in the majority of asthmatics; therefore, we believe that our results show no association between *C pneumoniae* and the exacerbation of asthma. Similar results were also obtained in the serologic testing of *Chlamydophila pneumonia*-specific antibodies in other large studies.^9–11^ In contrast, we were unable to any identify *M pneumoniae* in our investigations using PCR from the tracheal secretions of asthmatics in our study; therefore, we cannot confirm a link between *M pneumoniae* and the exacerbation of asthma. These inconsistent findings regarding a possible connection between atypical bacteria and asthma may be due to the difficulty in microbiologically identifying these organisms. These atypical bacteria are fastidious and difficult to grow on a bath culture under the usual conditions. Therefore, serological tests have been commonly used to detect antibodies against these types of germs for the detection of these organisms in most previous studies.^8^ Other recent studies have used PCR, as we did, to detect these atypical bacteria.^12–14^ New discoveries about other types of bacteria such as *S pneumoniae*, *Haemophilus influenzae*, *Moraxella catarrhalis*, or combinations of these organisms have been correlated with asthma.^15^ Although the data concerning those bacteria were collected in childhood asthma by Bisgaard et al, we found these microorganisms mainly in the non-specified followed by allergic asthma patients in our study. Klemets et al^16^ also reported that working asthmatics have an increased risk for infection with *S pneumoniae*. However, these bacteria were not detectable in the majority of patients with asthma in our study, and we did not find a statistically significant relationship between these bacteria and asthma in our study.

Although studies have not confirmed the causality of these bacteria for the development of asthma, these previously published data contribute to stimulating a lively discussion on the relationship between several types of bacteria to the risk and prognosis of asthma in the current medical literature.^17^ Hosoki et al^18^ have reported that *S aureus* can affect the course from the onset of asthma. *S aureus* induced the significant distribution of eosinophil-derived neurotoxin, the generation of superoxides, and the production of cytokines and chemokines. *S aureus* may be connected to the exacerbation of asthma and to eosinophilic inflammation in asthma. *S aureus* directly activates eosinophils via a platelet-activating factor receptor.^18^ We detected *S aureus* mainly in allergic asthmatics in our study. However, the association between *S aureus* and asthma cannot be confirmed by our results due to the small number of patients with *S aureus* infections in our study population. *H influenzae* is one of the most commonly isolated bacteria in chronic respiratory diseases. Respiratory infections caused by *H influenzae* and allergic respiratory diseases lead to chronic respiratory infections and features of neutrophilic asthma.^19^ Unsurprisingly, we were able to identify different types of *Haemophilus* bacteria in the airways of many of our study's patients. However, we were still unable to demonstrate an association between asthma and different species of *Haemophilus* bacteria, as it may simply be the case that species of *Haemophilus* often cause general bacterial respiratory infections in humans. Soriano et al,^20^ for example, often found *H influenzae*, *Haemophilus parainfluenzae*, and *M catarrhalis* in their study of adult patients with respiratory tract infections. Nishioka et al^21^ also reported an increased incidence of respiratory tract pathogens and antimicrobial susceptibilities of *S pneumonia*, *H influenza*, and *M catarrhalis* isolated in outpatients with respiratory infections. Respiratory infections were often blamed for exacerbations in COPD and asthma attacks. In the case of COPD, Philit et al identified the bacteria *H influenzae*, *S pneumoniae*, and *Pseudomonas aeruginosa*.

Among the viruses recovered, influenza virus and the *Respiratory syncytial virus* were dominant in this study. In cases of acute asthma, only 4 infectious agents were shown, namely *M pneumoniae*, *Influenza A*, *Respiratory syncytial virus*, and *Parainfluenza virus*.^22^ Earlier, respiratory infections were discovered by *P aeruginosa* in intensive care units.^23^ Perhaps these microorganisms generally cause respiratory infections. Infections caused by *P aeruginosa* belong to the class of opportunistic pathogens with frequent risk of infection in the hospital due to their high phenotypic diversity.^24^ Due to the current database's limitations, it is not yet known whether fungi and fungal allergens are relevant to the exacerbation of asthma.^25^ There is overwhelming evidence that the most common external triggers of asthma include fungi allergens.^26^ The diagnosis of a fungal allergy can be determined either by skin tests with antigens derived from fungi or by the measurement of specific IgE levels. A number of fungi, such as multiple species of *Aspergillosis*, are known to trigger asthma. Earlier researchers have found that other fungus species such as *Candida* are implicated in asthma.^27^ Whether this relationship is of causal importance for asthma cannot be determined based on our available data. Elevated levels of nitric oxide can be found in the breath of asthmatics, and increased levels of nitric oxide have been measured in the lower respiratory tract.^28–32^ Although the concentration of nitric oxide was not analyzed in all patients with asthma in our study, the sample examinations revealed no notably elevated levels of nitric oxide in this study population. Recent studies have demonstrated that, in non-invasive techniques such as induced sputum, a finding of FeNO provides results that are as reliable as those of the traditional gold-standard methods such as bronchoalveolar lavage or bronchial aspirate by flexible bronchoscopy for diagnosing asthma.^33^ Non-invasive techniques are much more pleasant for patients, and the analysis of FeNO can be used to diagnose asthma. Additionally, induced sputum is a useful biological medium for the diagnosis of asthma and the determination of harmful dust. Bronchoscopy with bronchoalveolar lavage has become an important diagnostic tool in asthma.^34^ Although not all patients were subjected to bronchoscopy in this study, the superiority of bronchoalveolar lavage and bronchial aspirate over sputum examination could not be determined for the microbiological analysis of tracheal secretions.

## STUDY LIMITATIONS

This study examined the microbiological growth of microorganisms from the tracheal secretions of patients with different types of asthma through various techniques in one department of internal medicine. The present study examined patients with asthma during an episode of infection and also included asthmatics in an infection-free interval.

## CONCLUSIONS

We were neither able to identify an increased incidence of microorganisms in patients with various types of asthma during an episode of infection nor during infection-free periods. Based on the data in this study, we were unable to find an infectious etiology of asthma.
